# FTO protects human granulosa cells from chemotherapy-induced cytotoxicity

**DOI:** 10.1186/s12958-022-00911-8

**Published:** 2022-02-26

**Authors:** Rongli Wang, Wei Wang, Lijun Wang, Linnan Yuan, Feiyan Cheng, Xin Guan, Nini Zheng, Xinyuan Yang

**Affiliations:** 1 Department of Obstetrics and Gynecology, the First Affiliated Hospital of Xi’an Jiaotong University, 710061 Xi’an, China; 2 Department of Anesthesiology, the First Affiliated Hospital of Xi’an Jiaotong University, 710061 Xi’an, China

**Keywords:** FTO, POF, Menstrual-derived stem cells, BNIP3

## Abstract

**Background:**

Premature ovarian failure (POF) is a serious problem for young women who receive chemotherapy, and its pathophysiological basis is the dysfunction of granulosa cells. According to previous reports, menstrual-derived stem cells (MenSCs) can restore ovarian function and folliculogenesis in mice with chemotherapy-induced POF. Fat mass- and obesity-associated (FTO) was reported to be associated with oocyte development and maturation. FTO was decreased in POF and may be a biomarker for the occurrence of POF. Knockdown of FTO in granulosa cells promoted cell apoptosis and inhibited proliferation. But the relationship between FTO and ovarian repair was still unclear. This study was aimed at investigating the FTO expression level and the role of FTO in the MenSCs recovering the function of injured granulosa cells.

**Method:**

First, cisplatin was used to establish a granulosa cell injury model. Then, the MenSCs and injured granulosa cell coculture model and POF mouse model were established in this study to explore the role of FTO. Furthermore, gain- and loss-of-function studies, small interfering RNA transfection, and meclofenamic acid (MA), a highly selective inhibitor of FTO, studies were also conducted to clarify the regulatory mechanism of FTO in granulosa cells.

**Results:**

MenSCs coculture could improve the function of injured granulosa cells by increasing the expression of FTO. MenSCs transplantation restored the expression of FTO in the ovaries of POF mice. Overexpression of FTO restored the injured cell proliferation and decreased apoptosis by regulating the expression of BNIP3. Down-regulation of FTO got the opposite results.

**Conclusions:**

In the treatment of MenSCs, FTO has a protective effect, which could improve the viability of granulosa cells after cisplatin treatment by decreasing the expression of BNIP3. Meanwhile, FTO may provide new insight into therapeutic targets for the chemotherapy-induced POF.

**Supplementary Information:**

The online version contains supplementary material available at 10.1186/s12958-022-00911-8.

## Background

Premature ovarian failure (POF) is prevalent in 1–3% of women under 40 years old, and its features include the disordered ovarian function, the elevated gonadotropin hormone, and descended estrogen levels. There are many potential causes for this disease involving genes, autoimmunity, and iatrogenicity [1]. The most important and common causes of POF are chemotherapy [2]. Various studies have indicated that chemotherapy could cause small follicle depletion and even exhaustion of oocytes in the ovaries of young females with cancer, leading to POF [3, 4]. Cisplatin (Cis-diamminedichloroplatinum-II (CDDP)) is a widely used and effective chemotherapeutic agent for the treatment of cancers, including gynecologic malignancies [5, 6]. However, when used at higher dosages, injury may occur, such as nephrotoxicity [6], ototoxicity [7], and reproductive toxicity [2, 8, 9]. Previous studies reported that cisplatin caused POF during clinical usage [8, 9] and non-clinical investigation [10], and exposure of granulosa cells to cisplatin caused growth arrest, DNA damage, and increased apoptosis [11]. Therefore, the preservation of fertility and gonadal function has become an important issue for patients receiving chemotherapy of reproductive age. To overcome these side effects, many studies have been performed, and protective adjuvants have been developed [12, 13]. In particular, the therapeutic potential of stem cells attracted increasing attention [14–16]. Animal experiments have indicated that transplantation mesenchymal stem cell (MSC) can partially ameliorate the structure and function of injured ovaries [17, 18]. In POF mouse/rat models, intraovarian or intravenous injection of MenSCs reduced apoptosis and restored ovarian function [14, 16, 19]. DiI-labeled MenSCs were demonstrated to localize to granulosa cells of immature follicles [16]. Moreover, a recent clinical trial indicated that in poor ovarian responders, autologous MenSCs intraovarian injection could increase clinical pregnancy and live births rates [20]. Our previous study also indicated that MenSCs have reparative effects on cisplatin-induced premature ovarian failure mice. MenSCs transplantation reduced ovarian interstitium fibrosis and apoptosis of granulosa cells [3]. However, the regulation mechanism is still unknown.

RNA methylation on the sixth Natom of adenylate (m^6^A), one of the most abundant modifications on RNA, has captured the attention of researchers [21]. m^6^A can regulate gene expression in many physiological processes, and abnormal m^6^A modifications may lead to dysfunctions of RNA, which can further trigger diseases such as oocyte development disorders [22], metabolic diseases [23], and cancers [24]. m^6^A is a series of proteins composed of m^6^A methyltransferase (writers), m^6^A demethylases (erasers), and m^6^A-binding proteins (readers) [25]. They can add, remove, and recognize the m^6^A-modified site, which indicates that m^6^A RNA methylation is reversible and dynamic. It can be catalyzed by “Writers” and be erased by “Erasers”. “Writers” are a complex that consists of METTL3, METTL14, RNA binding motif protein 15 (RBM15), Wilms’ tumor 1-associating protein (WTAP), zinc finger CCCH domain-containing protein 13 (ZC3H13), vir like m6A methyltransferase associated protein (KIAA1429), and others, and involves in oocyte maturation [26, 27]. “Readers” are a series of YTH domain-containing proteins, such as YTHDF1, YTHDF2, YTHDF3, and YTHDC1 [28, 29], that are essential for early zygotic development and oocyte competence. YTHDF2 knockout mice are infertile and compared with littermates, YTHDF2 knockout mice have significantly smaller testes [30]. “Erasers” are consist of FTO (fat mass- and obesity-associated) and α-ketoglutarate-dependent dioxygenase AlkB homolog 5 (ALKBH5). FTO catalyzes the demethylation of m^6^A and causes multiple malformations and growth retardation in loss-of-function mutation [31]. In male germ cells, down-regulation of FTO suppresses spermatogonial proliferation [28]. During female oocyte development, the levels of RNA methylation are decreased [32]. The most recent studies found that abnormal FTO was related to oocyte maturation disorder [33]. A previous study has demonstrated that compared to the control group, the expression level of FTO in premature ovarian insufficiency (POI) patients and mouse models was significantly lower [33]. Knockdown of FTO in granulosa cells promoted cell apoptosis and inhibited proliferation [33]. These findings strongly suggested the potential role of FTO expression in ovarian function. However, little is known about whether MenSCs could restore the function of damaged ovaries by regulating the expression of FTO in granulosa cells. And we were also interested in the underlying molecular mechanism by which FTO affects the proliferation and apoptosis of granulosa cells.

To answer these questions, we designed an experimental methodology combining in vivo and in vitro models. They are POF mouse and coculture of injured granulosa cells with MenSCs, respectively. Our results showed that cisplatin-induced granulosa cell apoptosis via regulating the expression of FTO and MenSCs could restore the expression level of FTO. Furthermore, we identified that BNIP3 was a downstream target of FTO in mediating granulosa cell proliferation and apoptosis.

## Materials and methods

### Cell culture

Human ovarian granulosa cell lines KGN (Procell CL-0603) were kindly provided by *Procell Life Science & Technology Co., Ltd* (Wuhan, China). Cells were cultured in a T25 flask (Corning, New York, USA) containing DMEM/F12 (Hyclone, Logan, UT) with 10% FBS (SiJiqing, China) in an incubator at 37°C with 5% CO_2_. The medium was changed every two days. When the cells reached 90% confluence, they were detached by 0.25% trypsin-EDTA and plated in a 6-well/96well plate for the subsequent experiments, or passaged at a ratio of 1:3.

### Isolation of menstrual-derived stem cells (MenSCs)

This study was approved in advanced by the Ethical Committee of The First Affiliated Hospital of Xi’an Jiaotong University, and all the participants have written an informed content. MenSCs were isolated and cultured as our previous study described [3]. Briefly, collecting the menstrual blood from 6 healthy women (about 25–30 years old), then transferring the blood into a 50 ml centrifuge tube, which pre-contained 10 ml of phosphate-buffered saline (PBS), 100 U/ml penicillin, 100 mg/ml streptomycin, amphotericin B (0.25 mg/ml), and ethylenediaminetetraacetic acid (EDTA) (2 mM) (Gibco, Grand Island, NY, USA). Ficoll-Paque Plus (GE Healthcare Amersham, UK) was used to separate and purify the MenSCs. Finally, cells were culture in DMEM/F12 (Hyclone, USA) with 10% fetal bovine serum (FBS) (Sijiqing, China), in a T25 flask (Corning, New York, USA) at 37 °C in 5% CO_2_.

### Establishing granulosa cell injury model and MenSCs-injured granulosa cells co-culture model

For the granulosa cell injury model, KGN was removed from the T25 flasks and seeded in a 96-well plate at a density of 1 × 10^4^. CDDP (Sigma–Aldrich, St. Louis, MO) was used to make the granulosa cell injury model. After overnight culture, removed the medium and replaced the fresh medium contained CDDP (0, 1, 5, 10, 15, 20 µM). Then the cells viability was measured by CCK-8 assay (Dojindo, Shanghai, China) at day 1, 2, and 3 according to the manufacturer’s instructions. Then the 50% inhibitory concentration (IC50) was chosen for the injury model in the later experiments.

For the MenSCs-injured granulosa cells co-culture model, we need to pre-seed the KGN (2 × 10^5^) in a 6-well plate and incubate for 24 h. Then, the CDDP was added for another 48 h. Finally, a 6-well transwell insert (6.5-mm polycarbonate membranes with pores 4.0 μm in size, Corning, USA) was applied. A total of 6 × 10^5^ MenSCs were resuspended in 500 µL complete culture medium and seeded in the upper chamber with 2 × 10^5^ injured granulosa cells resuspended in 1 ml complete culture medium in the lower chamber. After cells were cocultured for 48 h at 37°C in a 5% CO_2_ incubator, the cells in the lower chamber were harvested (supplement).

### Experimental animals, POF model establishment

To establish the POF models, C57BL/6 female mice, aged 6-8weeks, were purchased from Beijing Vital River Laboratory Animal Technology Co., Ltd. All experimental procedures were approved by the Ethical Committee and the Institutional Animal Care and Use Committee of Xi’an Jiaotong University. All animals were housed in a relatively stable environment with a cycle of lights on at 8 a.m. and off at 8 p.m., maintained room temperature (21–25 ℃) with water and food available ad libitum.

To establish the POF model, mice were injected with CDDP (2 mg/kg) intraperitoneally for 7 consecutive days, according to our previous study [3].

### Experimental design

#### Experiment 1

To demonstrat the protective effects of MenSCs transplantation on POF ovarian function, the POF mice were divided into two groups and administered either treatment of MenSCs (passage 3–5, 200 µl cell suspensions containing 2 × 10^6^) by tail vein injection (MenSCs treated group, *n* = 15) or an equal volume of the medium (POF group, *n* = 15) on day eight. There are equal numbers of mice treated with 0.9% normal saline as a control group (*n* = 15). The animals were euthanized by cervical dislocation after 7 days of treatment. And the serum and ovaries were collected. We have recorded the bodyweight of each mouse every day.

#### Experiment 2

To explore the effects of FTO inhibitor MA [34] on POF ovarian function, the mice were randomly divided into 4 groups: (1) DMSO group (dimethyl sulfoxide, a solvent of MA, *n* = 15); (2) MA group (*n* = 15); (3) cisplatin group (*n* = 15); (4) cisplatin + MA group (*n* = 15). The POF models were established as mentioned earlier. The MA was administered simultaneously with cisplatin injection at the dose of 10 mg/kg. After 7 days of treatment, the animals were euthanized by cervical dislocation, and its ovaries were collected. During the experiment, we recorded the bodyweight of each mouse every day.

### Enzyme Linked Immunosorbent Assay (Elisa)

Fourteen days after successful establishment of the model, the blood collected from the excised eyeball was transferred to a tube for centrifugation at 3000 r/min for 10 min at 4 ℃, after which serum was obtained. Then, mice Elisa panel kits (Meimian Biotechnology, Jiangsu, China) were used to measure serum oestradiol (E2), FSH, and AMH levels according to the kit instructions. (Experiment 1)

### Hematoxylin-eosin staining

The ovarian tissues were fixed in 4% paraformaldehyde for 24 h [35] and then embedded in paraffin, finally cut into a 5-µm serial section. Then the tissue sections were rehydrated by incubating in xylene and subjecting to an alcohol gradient of 100%-70%. After deparaffinization, the section was stained with hematoxylin and eosin (HE).

### Immunohistochemistry

Immunohistochemistry (IHC) was performed as previously described [36]. The ovarian tissues of mice were fixed in 4% formaldehyde and paraffin-embedded using standard procedures. First, consecutive 4-µm sections were cut, deparaffinized with xylenes, rehydrated, and retrieved the antigen in sodium citrate solution (pH 6.0) for 20 min. Then the slides were treated with 3% hydrogen peroxide to quench the endogenous peroxidase and blocked with 1% bovine serum albumin for 30 min to block the nonspecific binding. Next, tissue sections were incubated with primary antibody anti-FTO (Abcam, Cambridge, ab126605, USA; 1:150) overnight at 4 ℃. Finally, wash the slides with PBS three times and incubate the slides with HRP-conjugated goat anti-rabbit IgG secondary antibody (1:1000, Santa, Cruze) for another 30 min. Finally, a 3, 3-diaminobenzidine tetrahydrochloride (DAB) (Beyotime, Wuhan, China) substrate kit was applied to detect peroxidase reactivity. According to the manufacturer instructions, we prepared DAB peroxidase substrate in 5 ml ddH_2_O in a glass vial. Then, drop the DAB substrate on top of the slides and watch the brown staining. Dip slides into ice plus tap water to stop the reaction and rinse under cold tap water for 5 min.

### Plasmids and small interfering RNA transfection

Overexpression and inhibition of FTO were achieved by transfection of pCAG-FTO (Miaoling, Wuhan, China) and FTO small interfering RNAs (siRNAs) (Ribobio, Guangzhou, China) separately. The empty vector for the plasmids and siRNAs were also included. The transient transfection of KGN was performed using Lipofectamine 2000 reagent (Invitrogen, Carlsbad, CA) according to the manufacturer’s instructions. In brief, a total of 2 × 10^5^ cells per well were seeded into a 6-well plate. Until 60–80% confluence, 3.0 µg vector DNA or 50nM siRNA were transfected by 3.0 µL Lipofectamine 2000 per well for 6 h, then the medium was switched to fresh DMEM/F12. The overexpression and knockdown efficiency of the target gene was measured by qRT-PCR and western blotting.

### Western blotting analysis

Cells were harvested after 48 h incubation. The total protein was extracted by RIPA buffer, which was pre-added with protease inhibitor cocktail and PMSF. BCA kit (Beyotime, China) was used to detect the protein concentration. 30 µg of protein was subjected to 10% SDS - PAGE gels and then transferred polyvinylidene difluoride (PVDF) membrane. The membranes were blocked in 0.1% TBST (Tris – HCl buffer saline with 0.1% Tween – 20) contained 5% skimmed milk. Then, these membranes were incubated with corresponding primary antibodies in 4℃ overnight. The primary antibodies are as follows: FTO (1:1000, ab126605 Abcam, USA), BNIP3 (1:1000, ab109362 Abcam, USA), BAX (1:1000, 50599-2-Ig, Proteintech, China), Bcl-2(1:1000, 12789-1-AP, Proteintech, China), β-actin(1:1000, 66099-1-Ig, Proteintech, China). The next day, these membranes were incubated with peroxidase - conjugated secondary antibodies at room temperature for 1 h. Finally, these membranes were visualized by enhanced chemiluminescence (ECL) procedure.

### RNA extraction and quantitative real-time PCR (qRT-PCR)

Total RNA from cells was isolated using RNAiso Plus (Takara, Japan) according to the reagent instruction. Gene primers used in this study were as follows:

FTO (forward, 5’ – CTTCACCAAGGAGACTGCTATTTC − 3’, reverse, 5’ - CAAGGTTCCTGTTGAGCACTCTG − 3’),

METTL3 (forward, 5’ – TTGTCTCCAACCTTCCGTAGT – 3‘, reverse, 5’ – CCAGATCAGAGAGGTGGTGTAG – 3’), METTL14 (forward, 5’ – ACCTTGGAAGAGTGTGTTTACGA – 3’, reverse, 5’ – TGTGAGCCAGCCTTTGTTCT – 3’),

WTAP (forward, 5’–TGTGCTGTGTAAGGGCATTCGTACTCATGC–3’reverse5’–ACTGGGCAAACTTGGCAGTCATAAACCCAC–3’),

ZC3H13 (5’ – AAAGGAGGTTTCACCAGAAGTG – 3’, reverse, 5’ – CGCTTCGGAGATTTGCTAGAC – 3’),

KIAA1429 (5’ – AAGTGCCCCTGTTTTCGATAG – 3’, reverse, 5’ – ACCAGACCATCAGTATTCACCT – 3’),

RBM15 (5’ – AGCCGCGAGTATGATACCG – 3’, reverse, 5’ – GCCCGAAGAATTTTTGGTGCTC – 3’),

YTHDF1 (forward, 5’ – AACAATGAGGGCGAACCAGT – 3’, reverse, 5’ – GACACACTGGAGCTGACCAA – 3’),

YTHDF2 (forward, 5’– TAGCCAACTGCGACACATTC – 3’, reverse, 5’ – CACGACCTTGACGTTCCTTT – 3’),

YTHDF3 (forward, 5’ – TGACAACAAACCGGTTACCA – 3’, reverse, 5’ – TGTTTCTATTTCTCTCCCTACGC – 3’),

YTHDC1 (forward, 5’ – TCATCTTCCGTTCGTGCTGT – 3’, reverse, 5’ – TACAGGGAGCGTGGACCATA – 3’),

GAPDH (forward, 5’ – AAAATCAAGTGGGGCGATGCT – 3’, reverse, 5’ - TGGTTCACACCCATGACGAAC),

BNIP3 (forward, 5’ – TGAGTCTGGACGGAGTAGCTC – 3’, reverse, 5’ – CCCTGTTGGTATCTTGTGGTGT – 3’),

BAX (forward, 5’ – AGTGGCAGCTGACATGTTTT – 3’, reverse, 5’ – GGAGGAAGTCCAATGTCCAG – 3’),

Bcl-2 (forward, 5’ – TTCCACGCCGAAGGACAGCG – 3’, reverse, 5’ - GGCACTTGTGGCGGCCTGAT – 3’).

Real-time quantitative PCR was performed using SYBR Premix ExTaq™ (Takara) on a StepOne Real – Time PCR System (7300 Real – Time PCR system, Applied Biosystems, USA). The reaction conditions were as follows: 95 ℃ for 30s, 40 cycles at 95 ℃ for 15s, 60 ℃ for 30s, and extension at 72 ℃ for 60s. The target genes relative expression levels were analyzed by the 2^−ΔΔCt^ method.

### Cell counting kit-8 (cck-8) assay

The cell proliferation was determined by using the Cell Counting Kit-8 (CCK8) assay [37]. In brief, 1 × 10^4^ KGN cells per well were plated into a 96-well plate. Then, cells were cultured at 37 ℃ with 5% CO_2_ for 24 h. After the different treatments, 10 ul of CCK8 reagent (Dojindo, Kumamoto, Japan) and 90ul culture medium were added per well and incubated at 37 ℃ for 2 h. Finally, measured the absorbance at 450 nm at 24 h, 48 h, and 72 h.

### EdU labeling

To assess the KGN proliferation, 5-ethynyl‐2‐deoxyuridine (EdU) Apollo567 kit (Ribobio Co., Ltd.) was used. First, KGN (1 × 10^4^) were cultured in a 96-well plate and incubated with EdU (1:1000, 50 µmol/L) for 2 h. Then, KGN was fixed with 4% formaldehyde for 30 min at room temperature, followed by incubation with glycine (2 mg/ml) and PBS for 5 min respectively. Next, permeabilized the cells in 0.5% Triton X‐100 (100 µL) for 10 min and added the Apollo® reaction cocktail (100 µL) for 30 min under light‐shading conditions at room temperature. Finally, 4′,6′‐diamidino‐2‐phenylindole (DAPI) was used to counterstain the nuclei for 30 min at room temperature. After 3 times washes with PBS, the images were acquired using fluorescent microscopy (Olympus, Japan).

### Flow cytometry analysis

Annexin V – FITC Apoptosis Detection Kit (BD Biosciences, CA, USA) was used to assess the KGN cell apoptosis. Briefly, KGN was harvested and washed in ice-cold PBS. Then, resuspended the cells in binding buffer (200 µL) and added the Annexin V – FITC (5 µL) and propidium iodide (5 µL) in it at room temperature for 10 min (in darkness). Finally, added 300 µL binding buffer to each tube and the percentage of apoptotic KGN cells were analyzed using flow cytometer (FC 500, MCL, CA).

### Statistical analysis

All cell experiments were carried out in triplicate. The data were represented as mean ± SEM and statistical analysis was conducted with GraphPad Prism 5. Student’s t-test and one-way ANOVA were applied to compare the two experimental groups and multiple groups, respectively. And the data was analyzed with the observer blind to the treatment. *P* value <0.05 was considered statistically significant.

## Results

### Effects of cisplatin on KGN cell proliferation and apoptosis

**Fig. 1:**
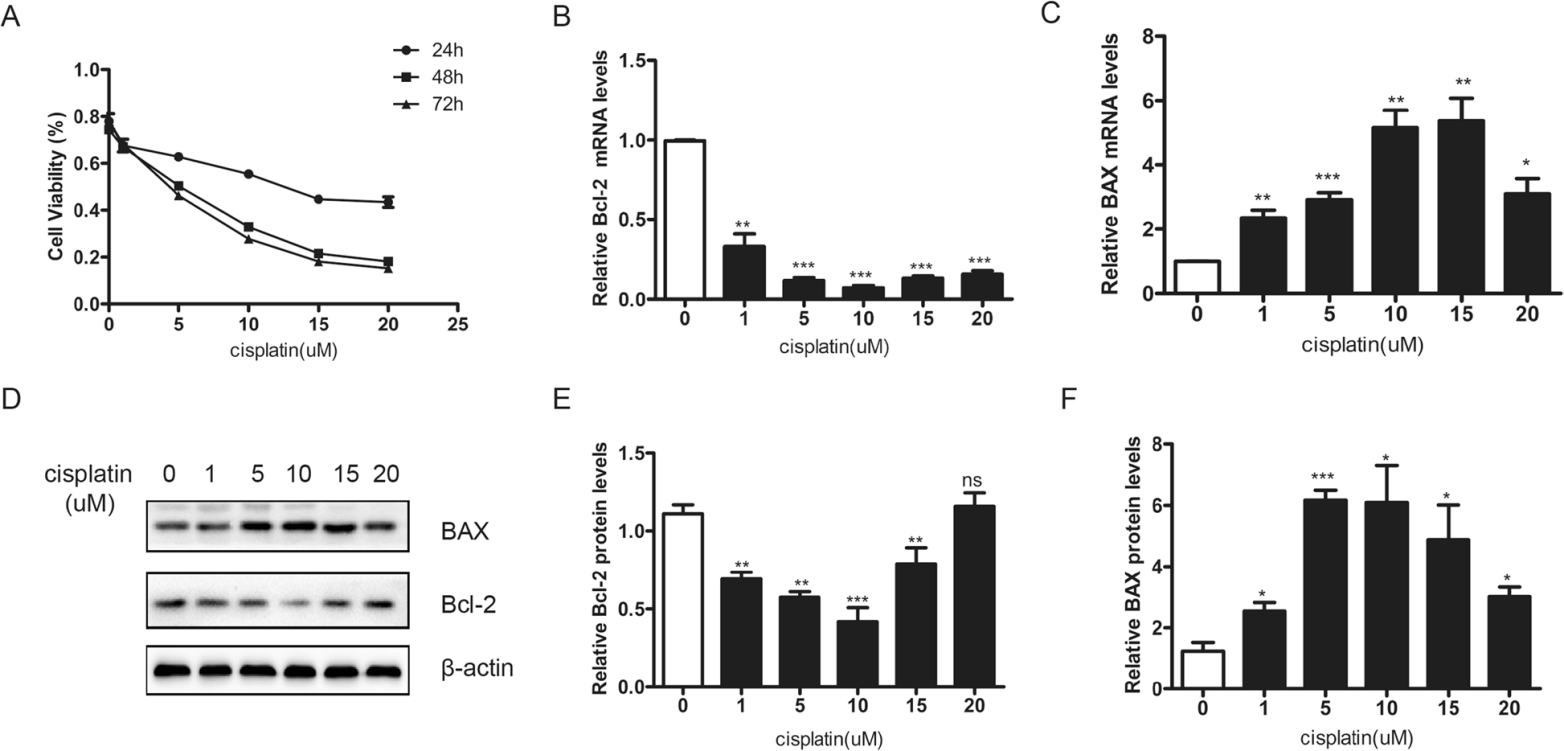
The effect of cisplatin on granulosa cells proliferation and apoptosis. **A** a CCK-8 assay was used to measure the effect of cisplatin on granulosa cell viability. Cisplatin inhibits granulosa cell proliferation rate in a dose-dependent and time-dependent manner. **B**-**F** qRT-PCR and western blotting showed that when the concentration of cisplatin was lower than 10 µM, as the concentration of cisplatin increased, the apoptotic ability of cells gradually increased, while the anti-apoptotic ability gradually weakened. When the cisplatin concentration was higher than 10 µM, the opposite result was obtained. One representative experiment of three independent experiments is shown. Data are shown as the mean ± SD from three independent experiments. β-actin served as the loading control. (*p < 0.05; **p < 0.01; ***p < 0.001 by Student’s t test. CCK-8: cell counting kit‐8)

To evaluate the effect of cisplatin on KGN cell viability, a CCK-8 assay was conducted. The results showed that cisplatin (0–20 µM) induced cell death in a dose-dependent and time-dependent fashion (Fig. 1A), and the 50% inhibitory concentration (IC50) (half-maximal inhibitory concentration) was obtained by treating cells with 10 µM cisplatin for 48 h. Then, we detected the mRNA and protein expression levels of BAX and Bcl-2 and found that when the concentration of cisplatin was lower than 10 µM, as the concentration of cisplatin increased, the apoptotic ability of cells gradually increased, while the anti-apoptotic ability gradually weakened. When the cisplatin concentration was higher than 10 µM, the opposite result was obtained (Fig. 1B-F). We speculate that when the concentration of cisplatin is more than 10 μm, the remaining cells produce a self-protective ability to resist cisplatin injury, resulting in the enhancement of their anti-apoptosis ability. We cannot exclude the possible existence of other mechanisms that explain this resistance to cisplatin injury. Finally, we used the KGN cell culture with cisplatin 10 µM for 48 h as the granulosa cell injury model.

### Treatment with MenSCs or conditioned medium improved injured granulosa cell function

**Fig. 2:**
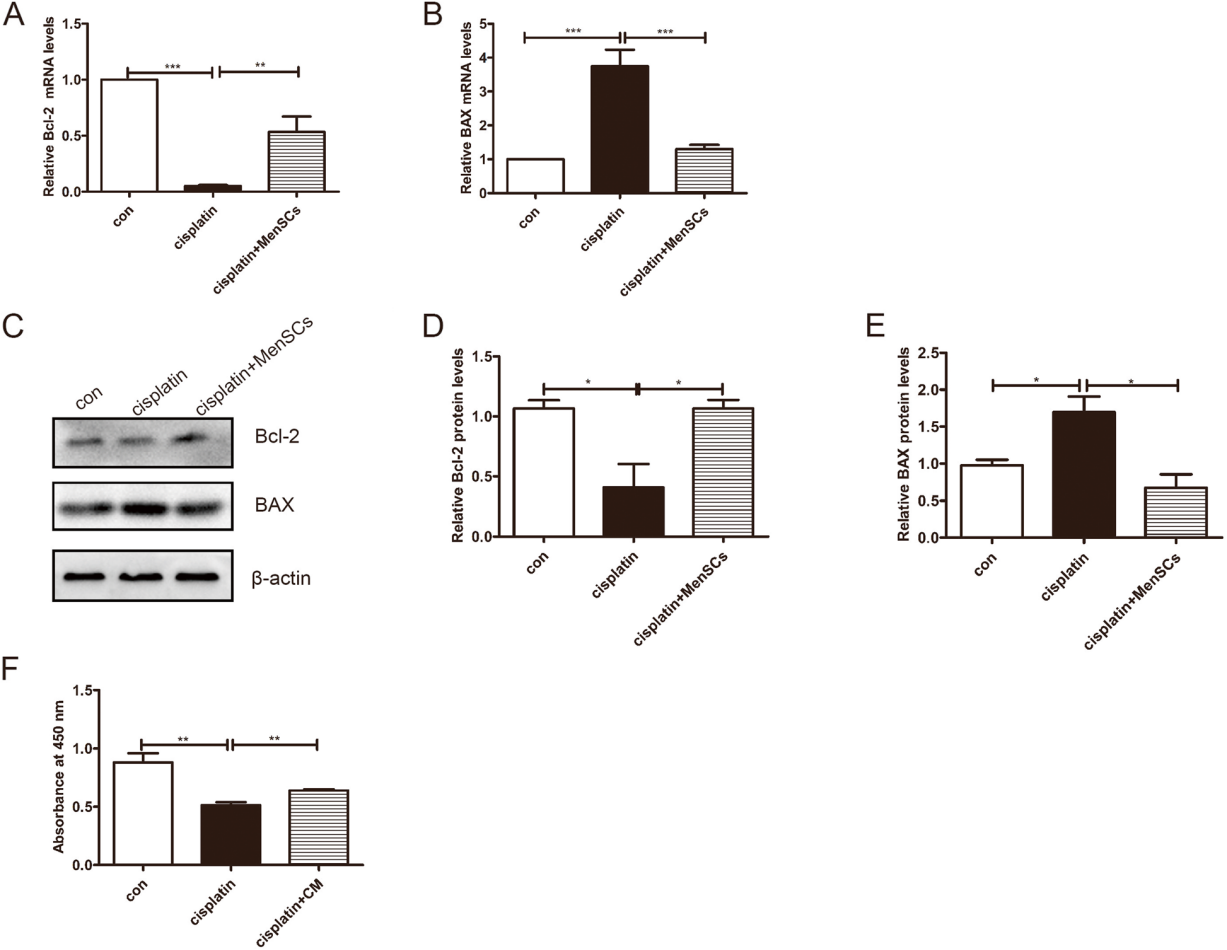
Effects of MenSCs and conditional medium (CM) on injured granulosa cells apoptosis and proliferation in vitro. **A**-**B** qRT-PCR and **C**-**E** western blotting indicated that MenSCs coculture with the injured granulosa cells could improve the injured granulosa cell anti-apoptosis ability by increasing the expression of Bcl-2 and decreasing the expression of BAX at the transcriptional and protein levels. One representative experiment of three independent experiments is shown. **F** A CCK-8 assay was used to measure the effect of condition medium (CM) obtained from the MenSCs on the in injured granulosa cells’ proliferation. The results showed that the CM could promote the injured granulosa cell viability significantly. β-actin served as the loading control. The results represent the mean ± SEM from three independent experiments. Student’s t test. (*P < 0.05, **P < 0.01, ***P < 0.001)

To examine the effect of MenSCs on injured granulosa cells, a cell coculture model was used to mimic the MenSCs-injured granulosa interplay in vitro. Compared with the cisplatin treatment alone, the anti-apoptotic ability of MenSCs cocultured injured granulosa cells was enhanced. As shown in (Fig. 2A-E), the mRNA and protein expression levels of Bcl-2 improved, while those of BAX decreased. Conditioned medium was collected from MenSCs. When MenSCs reached 80% confluence, the medium was switched to serum-free medium, and the cells were cultured for another 48 h. The CCK-8 assay showed that the conditioned medium could promote the injured granulosa cell viability (Fig. 2F). These results demonstrated that MenSCs promoted cisplatin-induced injured granulosa cell proliferation and decreased apoptosis.

### MenSCs transplantation increased weight and improved injured ovarian function

**Fig. 3:**
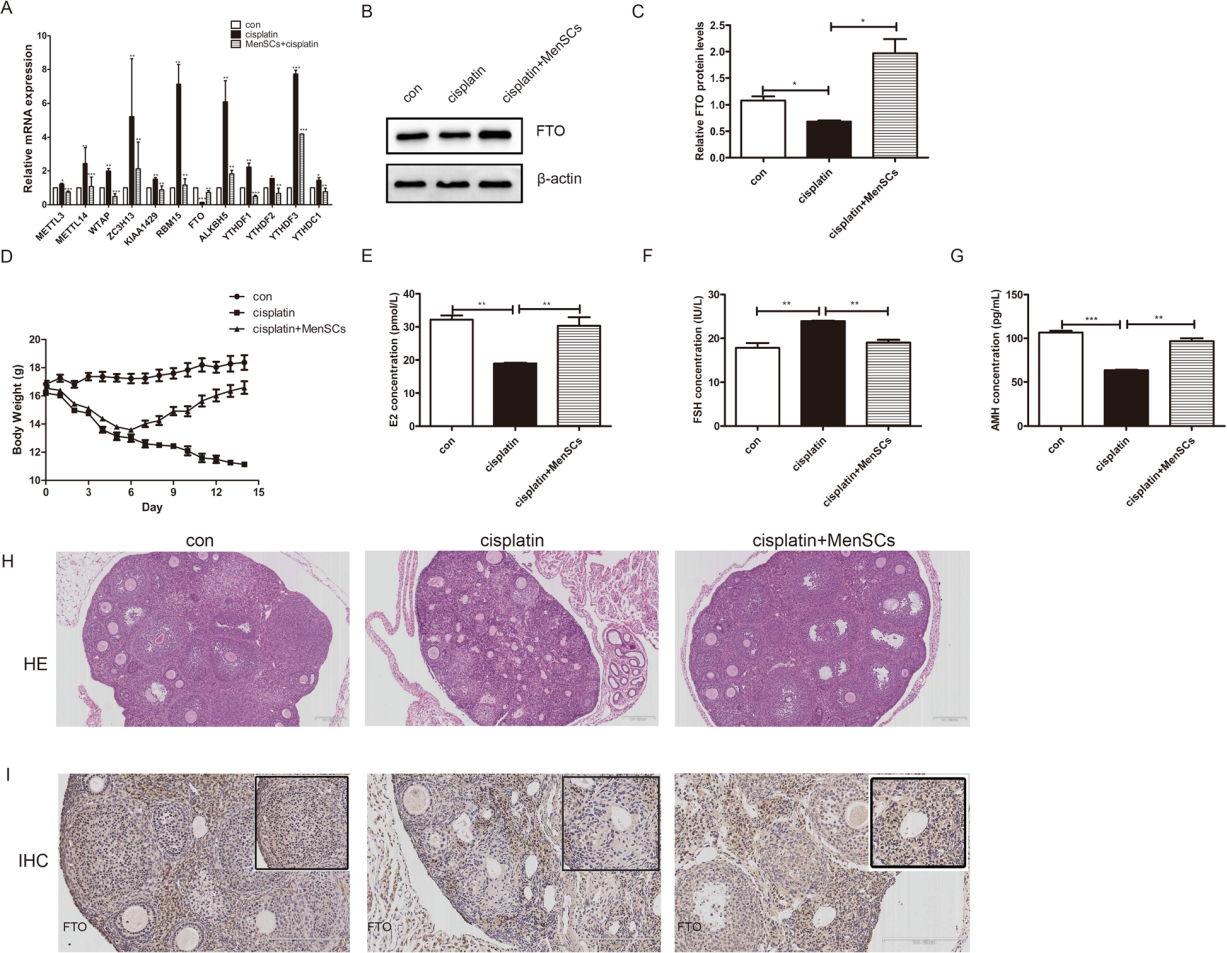
MenSCs restored the expression of FTO in injured granulosa cells and POF mice. **A**-**C** qRT-PCR and western blotting results showed that FTO expression was decreased in injured granulosa cells, and MenSCs co-culture could restore it. The results represent the mean ± SEM from three independent experiments. **D**-**G** POF mice models and MenSCs transplantation were established, and changes in the body weight, serum levels of E2, FSH, and AMH in different groups were shown. The graphs show the mean ± SEM (n = 15 mice per group). **H** H & E staining showed the morphological, follicle numbers, and histopathological change of the ovary after different treatments. **I** IHC results showed the FTO expression was decreased in the POF group compared to the controls, while MenSCs transplantation could restore it. (n = 15 mice per group). Scale bars in the HE staining represents 8 × 200 μm, while in the IHC staining represents 15 × 200 μm. Student’s t test. (*P < 0.05, **P < 0.01, ***P < 0.001)

As we described previously, the following evaluation indices were used to assess whether MenSCs transplantation could improve the ovarian dysfunction caused by cisplatin: weight, number of follicles, and serum sex hormone [3]. As shown in Fig. 3D, body weight increased significantly in the MenSCs transplantation group compared with the POF group. Furthermore, compared with the POF group, the levels of E2 and AMH were higher, while the level of FSH was lower in MenSCs group (Fig. 3E-G). The follicle numbers were increased significantly in the MenSCs transplantation groups compared with the POF group (Fig. 3H). These results demonstrated treatment with MenSCs improved ovarian function of POF mice.

### MenSCs restored the expression of FTO in injured granulosa cells and POF mice

We analyzed the expression levels of m^6^A-related elements and found that compared those in the cisplatin group, the mRNA expression levels of METTL3, METTL14, WTAP, ZC3H13, KIAA1429, RBM15, ALKBH5, YTHDF1, YTHDF2, YTHDF3, and YTHDC1 were all decreased in the MenSCs coculture model group, while the expression of FTO was increased (Fig. 3A). The protein expression level of FTO was also detected (Fig. 3B-C). We further detected the expression levels of FTO in animal models and found that in the POF group, the FTO expression was decreased, and the MenSCs transplantation reversed this decrease (Fig. 3I). These findings suggest that identification of FTO may explain, at least in part, the mechanisms of MenSCs improving the POF ovarian function.

### Overexpression of FTO reversed the effects of cisplatin on granulosa cells proliferation and apoptosis

**Fig. 4:**
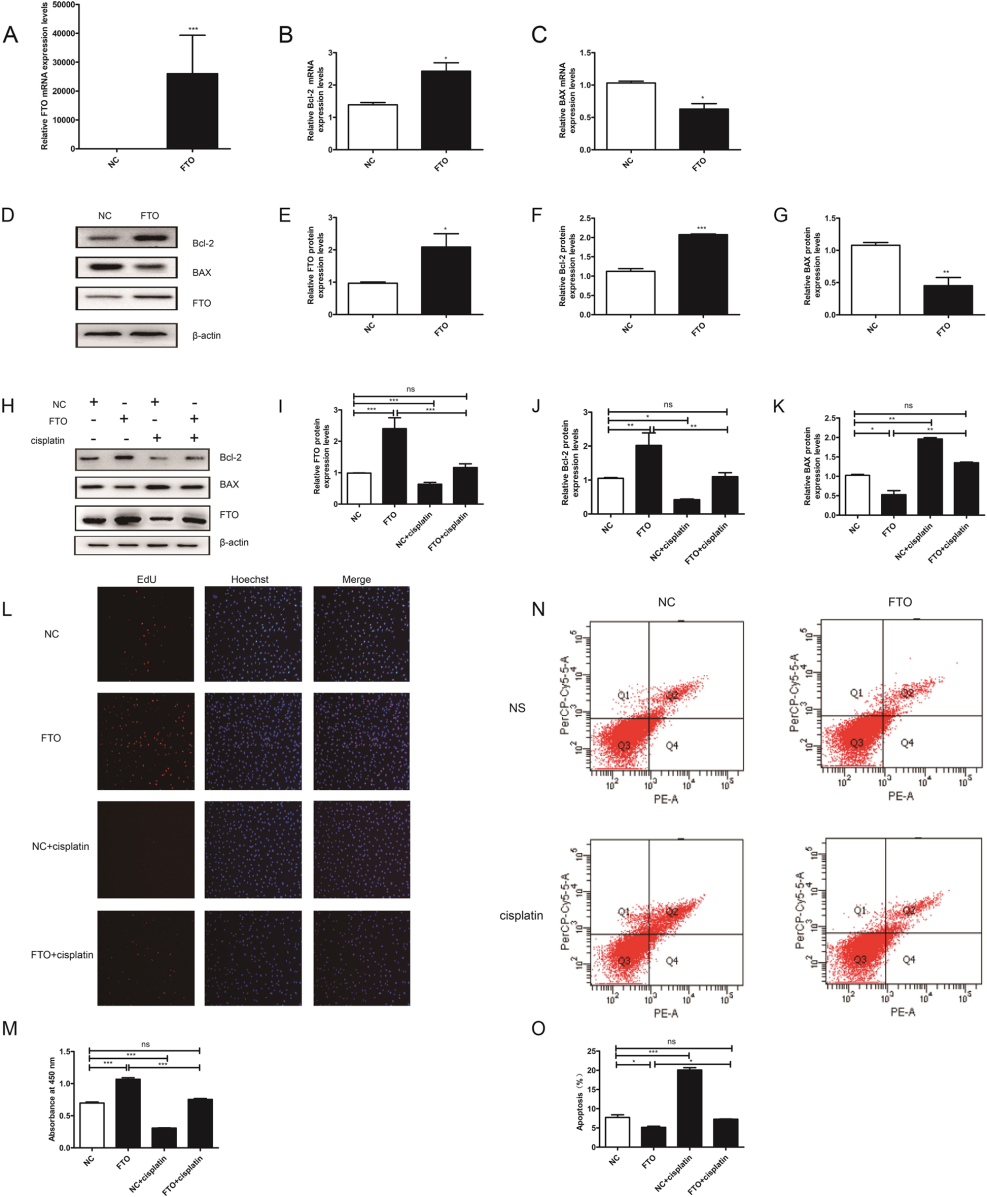
Overexpression of FTO attenuated cisplatin-induced granulosa cell apoptosis and promoted its proliferation. **A** The transfection efficiency of FTO was measured by qRT-PCR. **B**-**C** The mRNA expression of Bcl-2 and BAX in transfected granulosa cells was assessed using qRT-PCR and normalized to GAPDH. **D**-**G** The protein expression of FTO, BAX, and Bcl-2 was determined by western blotting with β-actin as a loading control in different groups. (H-K) Western blotting results showed that overexpression of FTO in injured granulosa cells could restore the cisplatin-induced change of FTO, Bcl-2, and BAX. **L**-**M** EdU labeling and cck-8 assay indicated that cells viability was inhibited in cisplatin-induced injured cells. However, transfection of FTO in injured cells, the viability was increased. **N**-**O** Flow cytometry assays were performed to determine the apoptotic rate in different groups. The results showed that cell apoptotic rate was increased in the cisplatin-induced injured cell, while co-transfection of FTO could restore it. One representative experiment of three independent experiments is shown. The results represent the mean ± SEM from three independent experiments. Student’s t-test and one-way ANOVA were applied to compare the two experimental and multiple groups, respectively. (*P < 0.05, **P < 0.01, ***P < 0.001, ns, no significance)

First, to explore the effect of FTO on granulosa cells viability, FTO was transiently transfected into granulosa cells, resulting in increased protein and transcript levels of FTO compared to those transfected with negative controls (NC) (Fig. 4A). CCK-8 and EdU assays showed that overexpression of FTO resulted in a significant increase in cell proliferation rate (Fig. 4L-M). qRT-PCR (Fig. 4B-C), western blotting (Fig. 4D-G), and flow cytometry – V/PE (Fig. 4N-O) staining showed that FTO could decrease granulosa cells apoptosis, resulting in a decrease in the expression level of BAX and an increased in the expression level of Bcl-2.

Then, we further investigated the effect of FTO on injured granulosa cells. Compared to the cisplatin-treated group, ectopic expression of FTO resulted in its up-regulation and restored the cisplatin-induced down-regulation of FTO in granulosa cells (Fig. 4H-K). In addition, CCK-8 and EdU assays showed that restoration of FTO expression prevented granulosa cells from decreasing the proliferation induced by cisplatin treatment (Fig. 4L-M). The rate of cell apoptosis was also tested, and the results showed that restoration of FTO decreased cell apoptosis. The expression levels of BAX and Bcl-2 also returned to the level of the NC (Fig. 4N-O). These results indicated that FTO is protective in cisplatin-induced damage.

### Down-regulation of FTO aggravates cisplatin-induced apoptosis

**Fig. 5:**
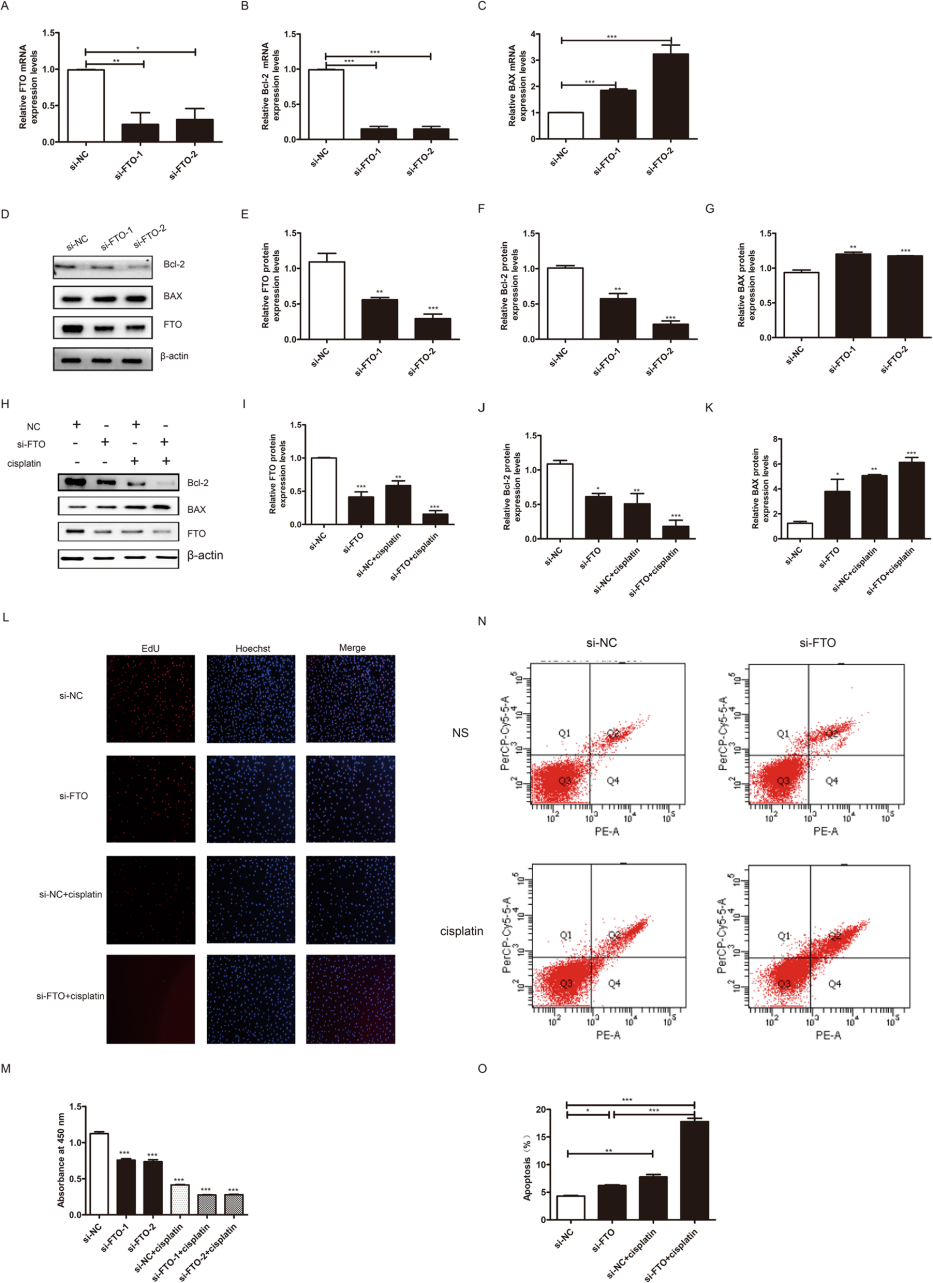
Down-regulation of FTO promoted injured granulosa cells apoptosis. **A** The transfection efficiency of si-FTO was measured by qRT-PCR. **B**-**C** The mRNA expression of Bcl-2 and BAX in transfected granulosa cells was assessed using qRT-PCR and normalized to GAPDH. **D**-**G** The protein expression of FTO, BAX, and Bcl-2 was determined by western blotting with β-actin as a loading control in different groups. (H-K) Western blotting results showed that inhibit FTO expression in injured granulosa cells could enhance the cisplatin-induced change of FTO, Bcl-2, and BAX. **L**-**M** EdU labeling and cck-8 assay indicated that cells viability was inhibited in cisplatin-induced injured cells, and co-transfection of si-FTO in injured cells could promote its effects. **N**-**O** Flow cytometry assays were performed to determine the apoptotic rate in different groups. The results showed that cells’ apoptotic rate was increased in the cisplatin-induced injured cell, and co-transfection of si-FTO could promote it. One representative experiment of three independent experiments is shown. The results represent the mean ± SEM from three independent experiments. Student’s t-test and one-way ANOVA were applied to compare the two experimental and multiple groups, respectively. (*P < 0.05, **P < 0.01, ***P < 0.001)

The effects of FTO knockdown on granulosa cell viability were also examined in this study. Transfection efficiency was confirmed by qRT-PCR. The mRNA level of FTO substantially decreased in the si-FTO group compared to the si-NC group (Fig. 5A). After 48 h of transfection with si-FTO, the cell proliferation rate was detected by CCK-8 and EdU assays. As shown in (Fig. 5L-M), the proliferation rate decreased significantly in the si-FTO group. Simultaneously, flow cytometry-V/PE staining showed that silencing of FTO in granulosa cells promoted cell apoptosis (Fig. 5N-O). FTO, BAX, and Bcl-2 expression levels were also detected by both qRT-PCR and western blotting. The expression of FTO and Bcl-2 decreased significantly in the si-FTO group, while the expression of BAX increased (Fig. 5A-G).

Furthermore, we investigated the effect of si-FTO on injured granulosa cells. The protein expression levels of FTO and Bcl-2 were lower and the expression level of BAX was higher in the si-FTO and cisplatin cotreated group compared to those in cisplatin-treated only group (Fig. 5H-K). The CCK-8, EdU, and flow cytometry-V/PE staining assays showed the same results (Fig. 5L-O). These results demonstrated that down-regulation of FTO aggravated the apoptosis of injured granulosa cells.

### FTO promoted granulosa cells proliferation and attenuated apoptosis may by targeting BNIP3

**Fig. 6:**
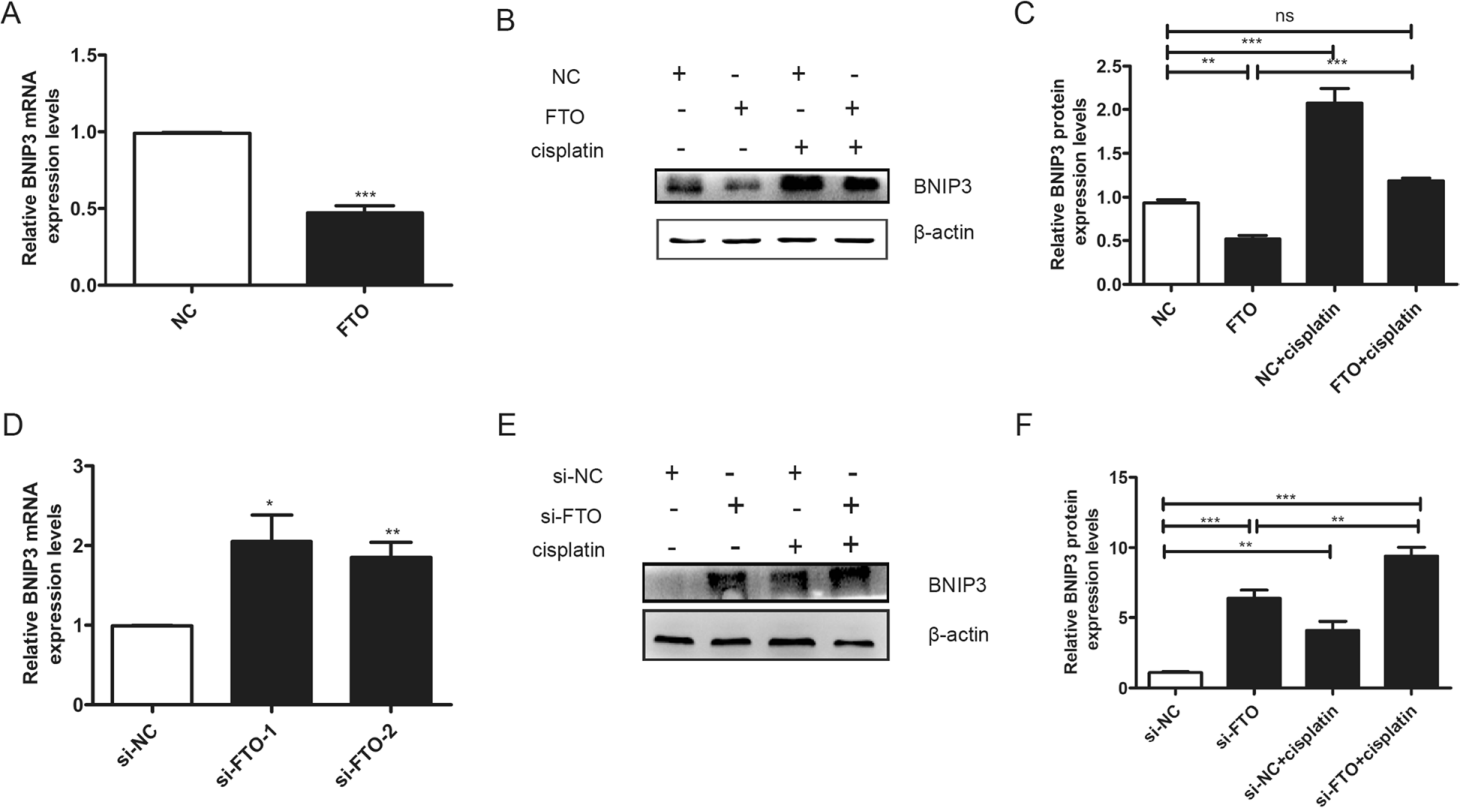
Overexpression of FTO attenuated injured granulosa cells’ apoptosis by downregulating the expression of BNIP3. **A** Compared to the NC group, the BNIP3 mRNA level was decreased in the FTO group. **B** The western blotting result showed that overexpression of FTO could reverse the expression level of BNIP3 induced by cisplatin. **C** qRT-PCR showed that downregulation of FTO increased the expression level of BNIP3. **D** The western blotting showed that downregulation of FTO promoted the expression level of BNIP3 induced by cisplatin. β-actin served as the loading control. One representative experiment of three independent experiments is shown. The results represent the mean ± SEM from three independent experiments. Student’s t-test and one-way ANOVA were applied to compare the two experimental and multiple groups, respectively. (*P < 0.05, **P < 0.01, ***P < 0.001, ns, no significance)

To verify BNIP3 as a downstream target of FTO, BNIP3 mRNA and protein expression levels were detected in granulosa cells. Compared to the NC group, overexpression of FTO decreased both the mRNA and protein levels of BNIP3 in normal granulosa cells (Fig. 6A-B). Furthermore, in the injured granulosa cells, we found that up-regulation of FTO reversed the changes in expression levels of BNIP3 induced by cisplatin to those of the NC group (Fig. 6B-C). However, down-regulation of FTO obtained the opposite results (Fig. 6E-G). These results demonstrated FTO considerably decreased BNIP3 expression at transcriptional and translation levels in granulosa cells. And downregulation of BNIP3 was involved in the FTO overexpression-induced proliferation of granulosa cells.

### An inhibitor of FTO (MA) promoted cisplatin-induced cytotoxicity in granulosa cells

**Fig. 7:**
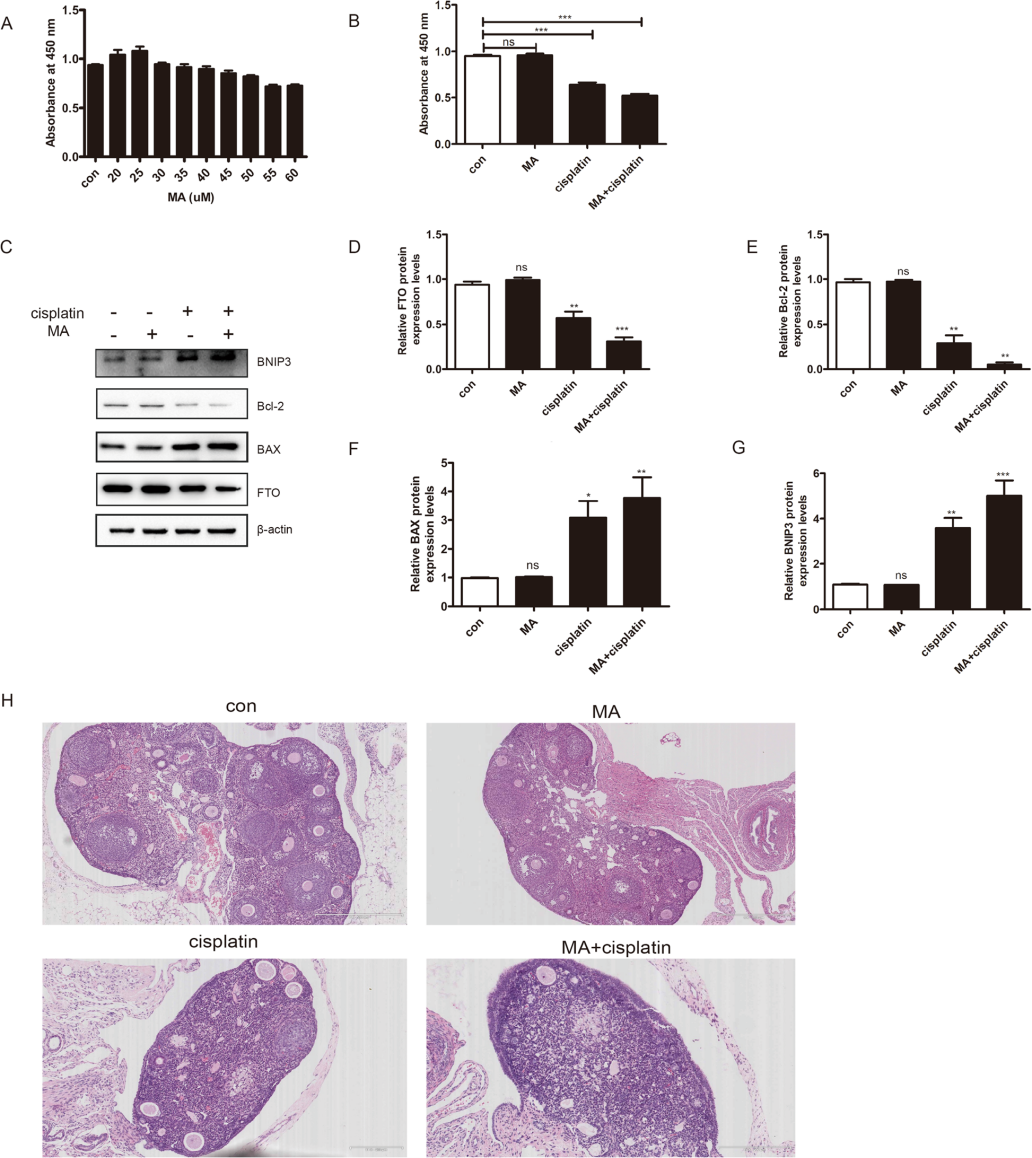
MA promoted cisplatin-induced cytotoxicity in granulosa cells, and cisplatin-induced injure in the ovary. **A** granulosa cells were incubated with different concentrations of MA for 48 h, the cell viability was measured by cck-8 assay. **B** granulosa cells were incubated with selected cisplatin in the presence or absence of MA for 48 h, and the cell viability was measure by cck-8 assay. **C**-**G** The western blotting showed that MA increased the cisplatin-induced BAX and BNIP3 expression and decreased the cisplatin-induced FTO and Bcl-2 expression. One representative experiment of three independent experiments is shown. The results represent the mean ± SEM from three independent experiments. one-way ANOVA was used to compare the multiple groups. **H** HE staining suggested MA promoted cisplatin-induced injure in the ovary. (n = 15 mice per group). (*P < 0.05, **P < 0.01, ***P < 0.001, ns, no significance)

Granulosa cells were treated with different concentrations of MA (0, 20, 25, 30, 35, 40, 45, 50, 55, 60 µM) for 48 h to determine the optimum treatment concentration of MA. Based on the results of CCK-8 assay, we chose 30 µM as the MA concentration for the subsequent experiments, which was the highest dose that showed no significant effect on cell death (Fig. 7A).

The cells and animals were divided into 4 groups, including the control group, MA- treated group, cisplatin-treated group, and cisplatin + MA-treated group, to explore the effect of MA on the cisplatin-induced injury. The results showed that compared to the control group and MA group, the cisplatin-treated group, and cisplatin + MA-treated group had significantly decreased cell proliferation rates (Fig. 7B). Furthermore, the cisplatin + MA treatment group showed aggravated the cell injury compared to the cisplatin-treated group (Fig. 7C-G). H & E staining showed that MA promoted the cisplatin-induced injury in the ovary (Fig. 7H). In the cisplatin + MA-treated group, the follicle numbers were significantly lower than cisplatin-treated alone. These results suggested that MA significantly increased cisplatin-induced granulosa cells apoptosis and ovarian damage.

## Discussion

POF is a serious complication of chemotherapy, and its incidence reaches 1/1000 in women under 30 years old, 1/250 in women under the age of 35 years old, and 1/100 in women under the age of 40 years old [38, 39]. Nowadays, the main treatment for POF is hormone replacement therapy [3]. However, it fails to resolve the fundamental problem, such as ovarian reserve and fertility restoration. Other strategies for female fertility preservation in the face of chemotherapy include embryo, oocyte, and ovarian tissue cryopreservation [40]. But transplantation of ovarian tissues may carry additional risks of reintroducing cancer cells [41]. Therefore, it’s urgent to explore other treatments that can prevent ovarian damage induced by chemotherapy.

Previous studies have demonstrated that MenSCs can restore ovarian function and decrease the granulosa cell apoptosis induced by chemotherapy agents [3, 14, 16, 42]; however, the regulatory mechanisms are still unknown. In this study, we first established a granulosa-injured cell model and MenSCs-injured granulosa coculture model and found that MenSCs could decrease granulosa cell apoptosis induced by cisplatin and that conditioned medium from MenSCs could promote injured granulosa cell proliferation. A previous study demonstrated that overexpression of FTO could decrease the cisplatin-induced cell apoptosis in acute kidney injury [43], and in POI patients and mouse models, the expression level of FTO decreased significantly compared with that in the control groups [33, 44]. Therefore, we wondered whether MenSCs promote injured granulosa cell proliferation and decrease apoptosis through up-regulation of FTO. The results showed that compared to the control group, FTO expression decreased in the cisplatin treatment group, while in the MenSCs-injured granulosa coculture group, the expression level of FTO increased. In addition, we established a POF mouse model and MenSCs transplantation, and the IHC results showed that the FTO expression decreased in the POF group and increased in the MenSCs transplantation group. These findings strongly suggested that FTO plays a vital role in recovering ovarian function.

To better understand the role of FTO in granulosa cell viability, gain- and loss-of-function studies were employed. The flow cytometry assay revealed that ectopic expression of FTO could attenuate cisplatin-induced apoptosis. CCK-8 and EdU assays showed that restoration of FTO expression prevented granulosa cells from cisplatin-mediated changes in cell proliferation. Knockdown of FTO by pharmacological (MA) or genetic (siRNA) constructs promoted cisplatin-induced apoptosis in granulosa cells. These findings suggested that cisplatin-induced granulosa cell apoptosis in an FTO-dependent manner.

At the molecular level, there are two pathways involved in granulosa cell apoptosis; death receptor and mitochondrial pathways [45]. In the mitochondrial pathways, the pro-apoptotic protein BAX can initiate apoptosis and accelerate follicular atresia [46], while Bcl-2 can act antagonistically on BAX and prevent cell apoptosis [47]. Then, we detected the expression levels of BAX and Bcl-2. qRT-PCR and western blotting showed that in the cisplatin treatment group, the expression level of Bax increased, while Bcl-2 expression decreased. Overexpression of FTO in the cisplatin group restored Bax and Bcl-2 to the levels observed in the NC group. However, downregulation of FTO in the cisplatin group promoted the Bax expression and decreased Bcl-2 expression. In summary, molecular and functional analyses confirmed that FTO plays a positive role in cisplatin-induced cell injury.

We further explored the downstream working mechanism of FTO. BNIP3, a member of the Bcl-2 family [48], is a target of FTO in breast cancer that mediates cell proliferation and apoptosis [49]. A previous study has demonstrated that BNIP3 was involved in the cisplatin-induced cell apoptosis [50]. We wondered whether BNIP3 acts as a target of FTO to participate in cisplatin-induced granulosa cell apoptosis. In our study, overexpression of FTO decreased BNIP3 expression at both the mRNA and protein levels. In addition, silencing FTO promoted BNIP3 expression. Furthermore, ectopic expression of FTO in cisplatin injured granulosa cells attenuated the increase in BNIP3 induced by cisplatin. Silencing FTO expression in injured granulosa cells produced the opposite results.

Previous studies have demonstrated that autophagy is involved in cisplatin-induced apoptosis in various cancers, such as pancreatic cancer [51] and lung cancer [52]. In hair cell-like HEI-OC1 cells, downregulation of FTO reduced reactive oxygen species (ROS) accumulation, inhibited apoptosis and the cisplatin-induced excessive autophagy, and protected and improved the viability of HEI-OC1 cells [7]. BNIP3, a downstream target of FTO [49], can act as an autophagy-related protein [53] and interact with LC-3 through the LC-3-interacting region to promote autophagy [54]. For example, in ovarian cancer cells, cisplatin-induced cellular autophagy was dependent on BNIP3 [50], and in the lung cancer cells, hypoxia augmented cisplatin-induced autophagy by suppressing the BNIP3 death pathway [55]. We speculated that in POF, overexpression of FTO inhibited cisplatin-induced granulosa cell apoptosis through BNIP3- mediated autophagy. We will explore this issue in our future studies.

There are many limitations to this study. Admittedly, our results would be more convincing if the expression of FTO was examined in ovary samples of POF patients and normal donors. However, we had no access to ovarian samples due to limitations in clinical treatment and ethics requirements. A previous study has reported that cisplatin-induced injured granulosa cells could promote bone marrow-derived mesenchymal stem cells (BMSCs) migration. BMSCs reduced injured cell apoptosis both in vivo and in vitro. However, in vivo, the migrated BMSCs did not locate in the follicles and corpus lutea [10]. We hypothesize that some factors must be secreted from the stem cells to protect injured granulosa cells. In this study, when the MenSCs were cocultured with injured granulosa cells, cell apoptosis decreased. Furthermore, we found that the expression of FTO increased in the injured cells. However, which factors secreted from the MenSCs promote the FTO expression is still unknown.

## Conclusions

In summary, our studies showed that MenSCs increase the expression of FTO to attenuate the cisplatin-induced granulosa cell apoptosis. The upregulation of FTO in granulosa cells decreased cisplatin-induced apoptosis by inhibiting the expression of BNIP3. Inhibition of FTO can further increase granulosa cell apoptosis induced by cisplatin. FTO may play a protective role in cisplatin-induced injured granulosa cell apoptosis.

## Supplementary Information

**Additional file 1.**

## Data Availability

The datasets used and/or analyzed during the current study are available from the corresponding author on reasonable request. All data generated or analyzed during this study are included in this published article.

## Abbreviations

POF: Premature ovarian failure
m^6^A: RNA methylation on the sixth Natom of adenylate
FTO: fat mass- and obesity-associated
MenSCs: menstrual-derived stem cells
MA: meclofenamic acid
CDDP: Cis-diamminedichloroplatinum-II
RBM15: RNA binding motif protein 15
WTAP: Wilms’ tumor 1-associating protein
ZC3H13: zinc finger CCCH domain-containing protein13
KIAA1429: vir like m6A methyltransferase associated protein
ALKBH5: α-ketoglutarate-dependent dioxygenase AlkB homolog 5
POI: premature ovarian insufficiency
IHC: Immunohistochemistry
PVDF: polyvinylidene difluoride
ECL: enhanced chemiluminescence
IC50: 50% inhibitory concentration
NC: negative controls
ROS: reactive oxygen species
