# Supplementary figures and images for: FTO protects human granulosa cells from chemotherapy-induced cytotoxicity
Source: Reprod Biol Endocrinol. 2022 Feb 26;20:39. doi: 10.1186/s12958-022-00911-8 (PMC8881882; doi:10.1186/s12958-022-00911-8)

The co-culture model is as follows:


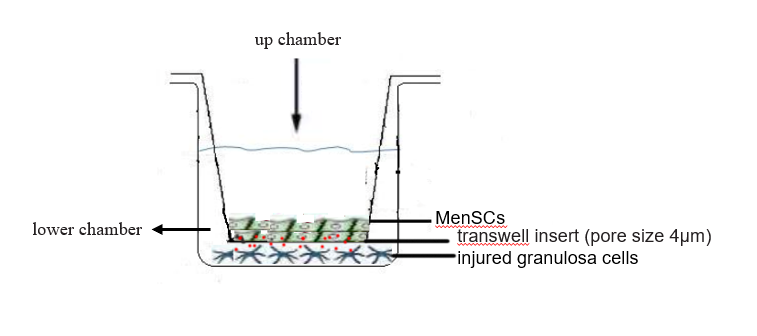

Supplement: Supplementary file 1 — Additional file 1. [file 12958_2022_911_MOESM1_ESM.docx]
